# High-Temperature Tensile Performance of Fused Filament Fabricated Discontinuous Carbon Fiber-Reinforced Polyamide

**DOI:** 10.3390/polym17131732

**Published:** 2025-06-21

**Authors:** Theodor Florian Zach, Mircea Cristian Dudescu, Paul Bere

**Affiliations:** 1 Department of Mechanical Engineering, Technical University of Cluj-Napoca, 103-105 Muncii Boulevard, 400641 Cluj-Napoca, Romania; theodor.zach@campus.utcluj.ro; 2 Department of Manufacturing Technology, Technical University of Cluj-Napoca, 103-105 Muncii Boulevard, 400641 Cluj-Napoca, Romania; paul.bere@tcm.utcluj.ro

**Keywords:** high-temperature-resilient composites, high-temperature tensile testing, infill orientation effect, discontinuous carbon fiber-reinforced polyamide

## Abstract

Fused filament fabrication of thermoplastic composites has grown exponentially owing to its efficiency, thereby meeting numerous engineering demands. However, these materials have limitations owing to their structural vulnerability to elevated temperatures. To address this drawback, this study aims to investigate the tensile behavior of 3D-printed composites in a broad thermal domain from ambient temperature to the crystallization point. For this purpose, a commercial high-temperature-resilient polyamide carbon fiber was selected. To assess the optimal bead configuration and application range, the methodology includes tensile testing of five infill orientations across the four principal thermal domains of the polymers. The results highlight different bead arrangements under constant thermal conditions and demonstrate how temperature effects the tensile performance at similar raster angles, as further correlated with fracture mechanism analysis via scanning electron microscopy. The key findings indicate that raster orientation has a minor influence compared to temperature change. In accordance with the literature, a significantly decreased strength and an abrupt increase in plasticity is observed above the glass transition temperature. Nevertheless, the material retains one-third of its ambient tensile strength at 150 °C, demonstrating its potential for high-temperature applications.

## 1. Introduction

Additive manufacturing of polymers via fused filament fabrication (FFF) entails the layered material extrusion of a filament onto a building plate to create predefined computerized shapes [1,2,3]. Initially developed as a rapid prototyping method, fused filament fabrication has expanded in market share by 25% annually, being primarily employed in the automotive, medical, and aerospace sectors and by the expiration of initial patents expanded to functional applications in consumer components [4,5,6]. Key advantages include material diversity including PLA (polylactic acid), ABS (acrylonitrile butadiene styrene), PET (polyethylene terephthalate), PA (polyamide), or advanced polymers such as PEEK (polyether ether ketone) [7,8,9]. By extending the range of available neat polymers, composites combine reinforcement materials (predominantly carbon or glass) with the aforementioned thermoplastic matrix resulting in an improved strength-to-weight ratio [10,11,12]. In comparison to traditional composite manufacturing techniques such as compression molding and pultrusion, FFF offers several advantages. It eliminates the need for dedicated tooling or storage facilities that pose chemical hazards, as well as complex post-manufacturing operations such as autoclaving. Consequently, FFF reduces both costs and lead times while maintaining increasingly satisfactory mechanical properties [13,14].

**Figure 1:**
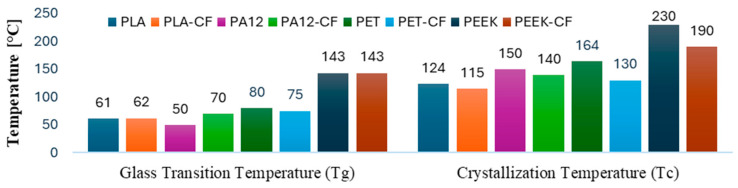
Thermal characteristics of thermoplastics and their composites. Adapted from [18,19,20,21,22,23,24,25,26].

Although thermoplastics and their composites exhibit satisfactory mechanical performance at ambient temperatures, their use is limited by heat-exposure softening. This phenomenon is attributed to an increase in the flexibility of cross-links within the semi-crystalline matrix structure when temperatures exceed the glass-transition temperature (T_g_). At this point, viscosity changes occur, and cross-chain rotation is initiated, continuing until the crystallization temperature (T_c_) is reached, where cross-links are substituted by van der Waals bonds. Upon reaching the melting temperature (T_m_), monomer rotation disrupts hydrogen bonds [15,16,17]. Considering their viscosity increase after T_g_, their structural viability is generally studied in environmental conditions. In the thermoplastic matrix commonly used in FFF (Figure 1), it can be observed that T_g_ is slightly elevated, while T_c_ is lower in composites compared to their pure thermoplastic counterparts. This is due to solid reinforcements, which act as crystallization initiation points.

The literature on tensile characterization at temperatures above ambient conditions is relatively limited in terms of materials or thermal domains. Grasso et al. [27] tested 3D-printed PLA in three raster configurations (0/90°, ±60°, and ±45°) at five temperatures (up to 60 °C). Their findings indicated that at room temperature (RT), the ±45° samples exhibited the maximum ultimate tensile strength (σ _UTS_) and Young’s modulus (E). However, above T_g_, the 0/90° orientation demonstrated the highest tensile results, although σ _UTS_ decreased by 80% and E halved, with the strain (ε) surpassing the limit value of 20%, irrespective of the orientation. Yavas [28] examined PEEK-CF with two infill orientations (0° and 90°) under three thermal conditions (up to 125 °C). At RT, the 0° specimens exhibited double their σ _UTS_ and a quarter increase in ε compared to their 90° counterparts. At 125 °C (still below the T_g_ of PEEK), the 0° orientation remained the strongest, σ _UTS_ decreased by 60%, and ε increased by 50%. These results can be better understood by correlating them with failure mechanisms. Following the FFF principle [2], fractures are produced by delamination of the adjacent rasters or layers. These interfaces can be classified as strong intra-laminar bonds (diffusion at similar temperatures) and weak inter-laminar cross-links caused by the thermal gradient between adjacent layers. In addition to these contact ruptures, cross-laminar brittle delamination (multi-level ruptures with neighboring beads attached) also leads to the failure of samples [29]. According to the literature, at RT, the 0° infill configuration exhibits a cross-laminar brittle material separation. Bead slipping occurs between the 0° and 90° raster orientation, resulting in combined intra-laminar decohesion opposed by slipping resistance. For the 90° infill, the load is resisted solely by intralayer adhesion forces. Alternating orientations (0/90° and ±45°) facilitate inter-laminar sliding, but the uniform load distribution limits bead extension in the latter configuration. Once the temperature rises, both the intra-laminar and inter-laminar bonding forces decrease owing to thermal creep, resulting in increased ductile behavior, which is more pronounced after reaching the matrix’s T_g_ [27,28,29,30].

To enhance understanding of the tensile characteristics of the 3D-printed composites discussed in the previous paragraph, this study investigates the tensile behavior of polyamide-carbon fiber across a broad thermal range up to the crystallization point of the matrix, including additional infill orientations beyond those reported in the literature, with the aim of establishing the optimal configuration and its thermal application range. Section 2 presents the characteristics of the selected material, the specimen preparation process and the high-temperature tensile testing methodology. Section 3 discusses the influence of infill configuration on tensile performance, followed by the thermal effect on the same bead orientation, correlating the results (σ _UTS_, ε, and E) with failure mechanism analysis via scanning electron microscopy (SEM). Section 4 provides a comprehensive comparison of the key findings with the literature and future directions.

## 2. Materials and Methods

### 2.1. Material Characteristics and Manufacturing Parameters

**Figure 2:**
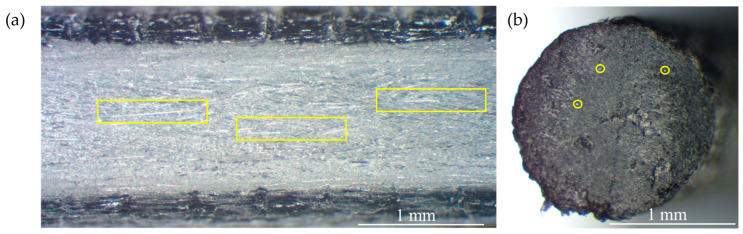
Microscopic images of the PAHT-CF filament: lateral view (**a**); cross-section (**b**).

To investigate the high-temperature tensile behavior of 3D-printed composites, a commercial heat-resilient polyamide-carbon fiber (PAHT-CF) supplied by Bambulab (Shenzhen, China) was selected. The starting point of this study was the Technical Data Sheet (TDS) of this material, which provided the ambient tensile properties listed in Table 1. Subsequently, the thermal application range was determined considering its heat deflection temperatures of 170 °C/194 °C (1.8 MPa/0.45 MPa). Moreover, the thermal characteristics of the material include a glass transition temperature (T_g_) of 70 °C, a crystallization temperature (T_c_) of 140 °C, and a melting point of 225 °C. To elucidate the discontinuous reinforcement distribution (15% mass content) in the raw material, microscopic examination at ×40 magnification was conducted. The filament presents discontinuous reinforcement in a mostly parallel orientation with the extrusion direction (yellow rectangles and circles in Figure 2), appearing in lighter shades, while the thermoplastic matrix is present in darker shades between the carbon fibers [26].

**Figure 3:**
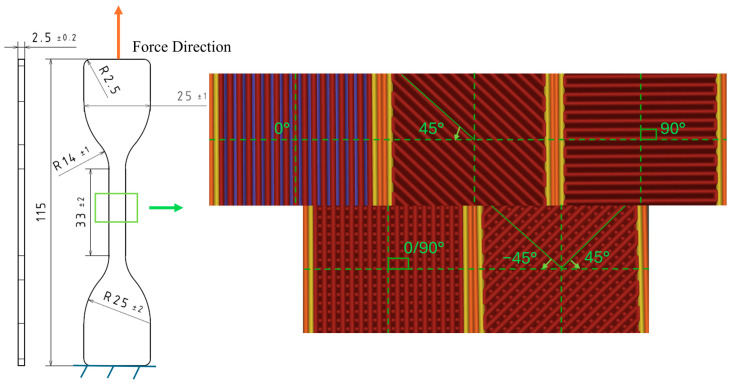
Specimen’s characteristics: dimensions in mm; infill orientations relative to the force.

Considering the available apparatus for the tensile characterization of PAHT-CF, the specimens conformed to ISO 23529 Type 1 (Figure 3) [31]. The specimens were manufactured in an enclosed Bambulab X1 Carbon FFF 3D printer (Shenzhen, China) [32] with a flat orientation, under humidity regulated below 10%, and preserved in a vacuum-sealed dehumidified container. The manufacturing parameters summarized in Table 2 were derived from the TDS of the material (identical to the specimens whose characteristics are presented in Table 1) and configured in Bambu Studio 2.1.1 software. The bead configurations identified in the literature are shown on the right-hand side of Figure 3. In addition to these infill angles, this study examined the 45° bead orientation to enhance understanding of its alternating counterpart (±45°).

### 2.2. High-Temperature Testing Procedure

To enable the broad tensile characterization of PAHT-CF, its thermal domains were defined. The first domain included tests below T_g_, representing the typical operating conditions for polymers: 25 °C for ambient applications and 50 °C (the T_g_ of pure PA found in the literature), chosen to examine the influence of reinforcement on the tensile behavior of the composite material. The second category encompassed the tensile investigation above T_g_ (100 °C is the midpoint between T_g_ and T_c_), whereas the third category evaluated the specimens above T_c_, at 150 °C, (capability of the tensile equipment). From the TDS, it can be concluded that temperatures above 150 °C result in abrupt strength decreases, as indicated by the proximity of the heat deflection temperatures provided by the supplier.

**Figure 4:**
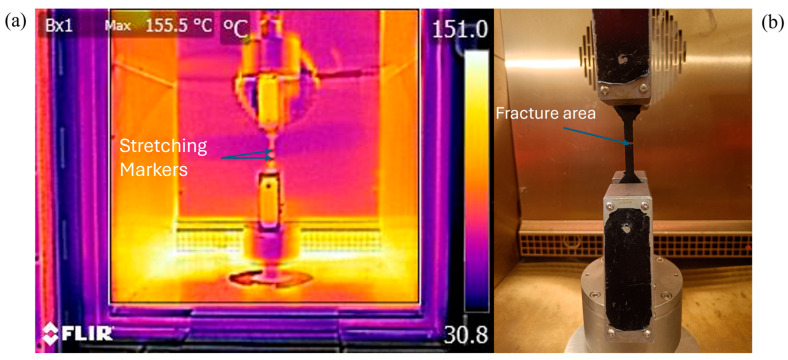
Specimen temperature monitoring (**a**); specimen after failure (**b**).

The high-temperature uniaxial tensile testing procedure, in accordance with ISO 37 [33] (the standard method for the available apparatus), is equivalent to ISO 527 [34] conditions used in the TDS. Tensile testing was performed using a 5967 Dual-Column Universal Machine (Instron, Norwood, MA, USA), utilizing a 2580-10KN/131510 force sensor and a 2663-901 extensometer from the same supplier, set to a 10 mm/s displacement. To ensure a uniform thermal distribution within the specimens, they were conditioned for 30 min at the corresponding testing temperature in a 3119-610 (Instron, Norwood, MA, USA) thermally controlled chamber. Conditions were permanently monitored by the sensor of the thermal chamber and checked using a FLIR E6 Pro (Wilsonville, OR, US) thermal camera, with an emissivity value of 0.95 (Figure 4a). Each sample configuration (infill orientation and testing temperature) included 5 specimens (totaling 20 sets/100 samples).

## 3. Results and Discussion

### 3.1. Influence of Infill Orientation on the Tensile Performance

The tensile results, given as median values for each set including the ultimate tensile strength (σ _UTS_), strain at failure (ε), and secant Young’s modulus (E), are presented relative to the 0° infill orientation (used as the reference), with standard deviation (Std.dev.) also provided. These results are correlated with the fracture mechanics of the area indicated in Figure 4b. The apparatus used for the SEM analysis was a JEOL IT200LA SEM (Akishima, Tokyo, Japan) with a ×75 magnification rate (optimal considering the nature of the material) and a 200 µm scale was utilized after the samples were gold coated to enhance the image quality. The images were interpreted based on findings in the literature [27,28,29,30].

**Figure 5:**
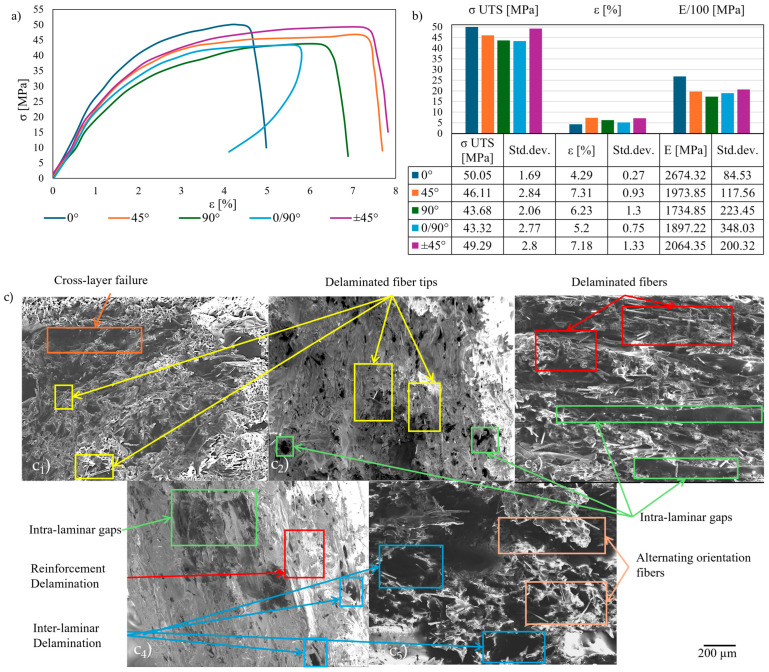
Tensile tests at 25 °C: stress–strain curves (**a**); tensile results chart (**b**); cross-section SEM of the fracture area (**c**): 0° (**c_1_**); 45° (**c_2_**); 90° (**c_3_**); 0/90° (**c_4_**); ±45° (**c_5_**).

The initial temperature examined was ambient (25 °C). The findings encompass stress–strain curves (Figure 5a), tensile results (Figure 5b), and SEM images of the fracture area (Figure 5c), which are interrelated. PAHT-CF exhibited plastic behavior, with the steepest gradient observed in the 0° samples, highlighted by a maximum σ _UTS_ of approximately 50 MPa and minimal strain. The SEM images revealed a brittle cross-laminar failure mechanism, in which the deformed, snapped reinforcement sustained the load. Stress–strain curves for the 45° and ±45° bead configurations demonstrated a reduced gradient, with strength values approximating the reference (particularly in the ±45° samples), but exhibited the highest strain values among the selected orientations. The maximum plasticity for 45° orientations was confirmed by plastic intra-laminar bead slipping towards the load orientation in the SEM images, showing a strained matrix with randomly oriented delaminated fiber tips. This phenomenon was mitigated by the alternating layer orientation, resulting in inter-laminar shear as the cause of fracture. The mildest gradient in the stress–strain curves, with the smallest σ _UTS_ and average strain values, was observed for the 90° and 0/90° bead configurations. The latter exhibited strain reduction post-failure, which was attributable to viscoelastic matrix relaxation and localized reinforcement retraction following load removal [35]. This is corroborated by intra-laminar bead delamination for the 90° layers, with deformed delaminated reinforcement for the 0° layers and inter-laminar shear for the 0/90° specimens. The σ _UTS_ decreased by 1.5–13.5% relative to the reference, with the lowest values observed in the 0/90° specimens. The strain increased by 20–70% relative to the reference, with a maximum observed in the 45° infill orientation. The 0° samples demonstrated the highest Young’s modulus values, with significantly lower values (22.5–35%) for the other configurations, and the minimum observed in the 90° samples. The tensile response and failure mechanisms are consistent with those reported in the literature.

**Figure 6:**
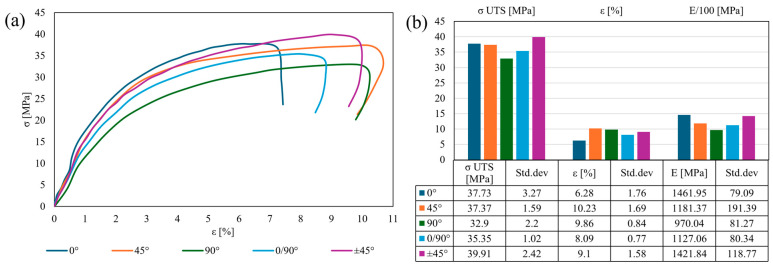
Tensile tests at 50 °C: stress–strain curves (**a**); tensile results chart (**b**).

The first thermal increment (50 °C), still under T_g_, displayed similar strain–stress profiles for almost all orientations (Figure 6a). The exception was the 0° infill orientation, with its brittle behavior indicated by the steep gradient of its curve, confirmed by the near-maximum σ _UTS_ and minimum plasticity (Figure 6b). The following stress–strain curves (45° and ±45° orientations) have a gradual inclination, overlapping until they reach 5% ε. The gradient of these profiles was confirmed by the highest σ _UTS_ (±45°) and the maximum ε among all orientations. The 0/90° orientation displayed an average plasticity between its components, with a steeper gradient than the 90° infill owing to its considerably lower strain values. The σ _UTS_ varied with orientation relative to the reference, showing an increase of 5% (±45°) and a decrease of 1–12.5%, with the lowest value observed in the 90° specimens. The strain decreased significantly with orientation relative to the reference by 30–62.5%, with a maximum value of over 10% for the 45° specimens. Conversely, post-failure strain reduction was observed for all orientations except the 0° infill, owing to matrix viscoelastic relaxation, which was further enhanced by thermal softening. The Young’s modulus decreased with respect to the reference from 2.75% (±45° specimens) to a maximum of 35% for the 90° infill.

**Figure 7:**
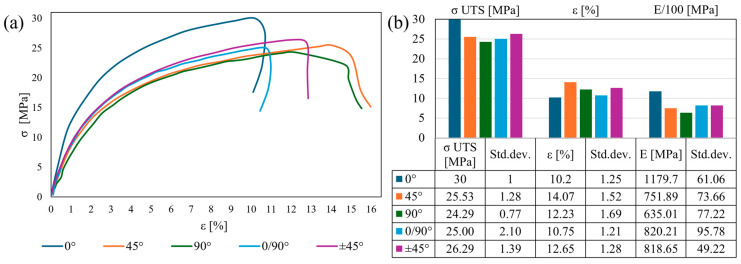
Tensile tests at 100 °C: stress–strain curves (**a**); tensile results chart (**b**).

The first temperature over T_g_ was 100 °C, which is the midpoint between T_g_ and T_c_, where the strain–stress curves (Figure 7a) presented a steep gradient for 0° specimens, confirming its relative brittle behavior compared to the other orientations. It was strengthened by its maximum σ _UTS_ and low plasticity (Figure 7b). Subsequently, for both the 0° and 0/90° specimens, a post-failure strain reduction was observed owing to the localized retraction of the reinforcement, which withstood the load [35]. The +45° stress–strain curve demonstrated relatively increased plastic behavior, as confirmed by its σ _UTS_ and ε values being amongst the highest. Close to this gradient, the 0/90° curve was differentiated by reduced σ _UTS_ and ε values. The highest plastic behavior, based on the stress–strain curves, was observed for the 45° and 90° infills, characterized by the lowest σ _UTS_ and highest ε among all orientations. Statistically, σ _UTS_ decreased substantially compared to the reference by 12.5–20% to reach its minimum in the 90° specimens. Moreover, the strain gradually increased from 5 to 40% to reach its maximum in the 45° specimens. Nevertheless, E decreased drastically from the 0° orientation by 30–50%, reaching its minimum in the 90° samples.

**Figure 8:**
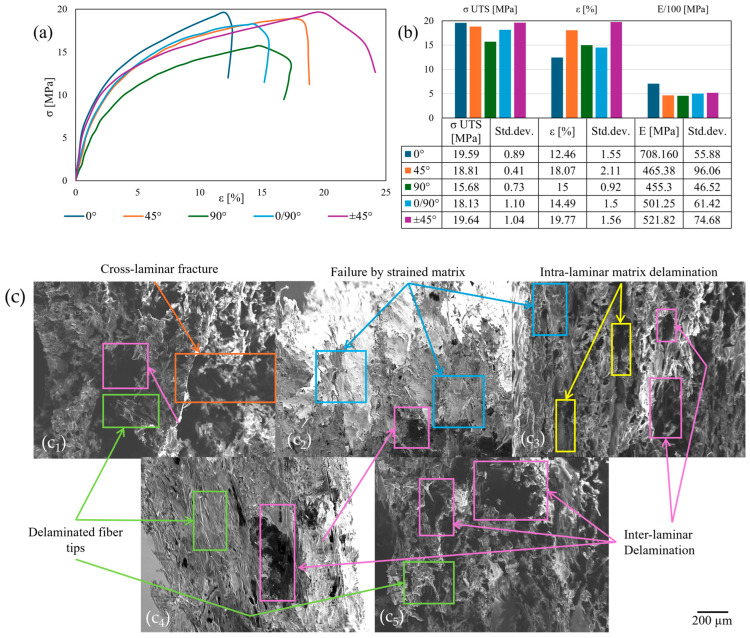
Tensile tests at 150 °C: stress–strain curves (**a**); tensile results chart (**b**); cross-section SEM of the fracture area (**c**): 0° (**c_1_**); 45° (**c_2_**); 90° (**c_3_**); 0/90° (**c_4_**); ±45° (**c_5_**).

The maximum testing temperature was established at 150 °C between T_c_ and the heat deflection temperature. The stress–strain curves, which are generally ductile (Figure 8a), along with the tensile results (Figure 8b) and fracture area analysis via SEM images (Figure 8c), exhibited the steepest gradient for the 0° specimens, attributable to the near-maximum ultimate tensile strength (σ _UTS_), slightly below 20 MPa, and the lowest plasticity. Although inter-laminar shear is present in all samples due to matrix thermal creep, the stress–strain profile is corroborated by a brittle cross-layer failure mechanism observed in the SEM images. The subsequent three stress–strain profiles (45°, ±45°, and 0/90° specimens) overlap until reaching their respective maximum σ _UTS_ values and differing in their ε values. In addition to inter-laminar delamination, the failure areas across these orientations reveal an overly strained matrix with embedded reinforcement, except for delaminated fiber tips in the 0° layers of 0/90° specimens. The variation in σ _UTS_ is attributed to the influence of reinforcement orientation, being lowest in the 90° layers of 0/90° samples and strongest due to the triangulation of ±45° specimens. The least steep gradient is observed in the 90° infill orientation, as confirmed by the lowest σ _UTS_, due to intra-laminar thermally softened matrix failure visible in the SEM analysis. Post-failure strain reduction is noted due to localized reinforcement retraction in the 0° and 0/90° specimens, and viscoelastic matrix relaxation in the 90° orientation, while an increase is observed in the ±45° infill due to inter-laminar failure caused by weaker and more plastic inter-layer bonds. The σ _UTS_ varied from a 1% increase, attributed to manufacturing variation visible in the standard deviation (0° and ±45°), to 20%, with the minimum observed in 90° samples. Strain increased relative to the reference orientation by 6.5–35%, with maximum values in the ±45° infill orientation. Young’s modulus reached its peak for the 0° infill, decreasing by 39–47.75% to a minimum in 90° specimens.

In summary, there was an alternation in the highest σ _UTS_ between the 0° and ±45° samples, although small gaps can be associated with manufacturing variations. The 45° samples followed, while the 90° raster orientation demonstrated the lowest values irrespective of temperature, except at 25 °C, where the 0/90° samples were lower, confirming that the tensile behavior of PAHT-CF is similar to that of PEEK-CF. The dispersion of the σ _UTS_ with orientation increased gradually from ambient temperature to stabilize at approximately 20% above T_g_. In terms of plasticity, 0° bead orientations displayed the lowest ε values irrespective of temperature, followed by 0/90° as the average of its alternating components, whereas 45° and ±45° alternated to obtain the maximum ε. The strain variation at 25 °C (70%) decreased substantially at 50 °C (45%) and then gradually reduced by 5% with each thermal increment. In terms of Young’s modulus, the 0° specimens exhibited the maximum values, followed by the ±45° samples, which showed an increasing difference after 50 °C, while the 90° configuration showed the smallest values under all thermal conditions. The T_g_ can be considered a turning point in E variation from lower dispersion (35%) below it to increased influence (50%) at 100–150 °C. Failure mechanisms at ambient temperature displayed alternation between brittle cross-laminar fracture (0°and 0/90°) and ductile intra-laminar breaking (45°, ±45°, and 90°), whereas above T_g_, ductile fracture was doubled by inter-laminar delamination. The ambient values for the strongest configuration (0° samples) were compared to the supplier data (Table 1), displaying lower values of 45% for σ _UTS_, 30% for E, and 50% for ε. These differences can be attributed to the post-manufacturing conditioning considered by the supplier for the tested samples. Overall, the tensile behavior of PAHT-CF confirms the data from the literature, where T_g_ can be considered a turning point for the orientation effect on tensile performance, although the stabilization for strain was observed at 50 °C (the T_g_ of pure PA12), indicating a reduced influence of reinforcement on the plasticity of 3D-printed composites.

### 3.2. Influence of Temperature on the Tensile Behavior for Constant Infill Orientation

The influence of temperature on tensile performance was evaluated for three primary infill orientations, specifically, alternating between the strongest and least resistant orientations, with the remaining orientations being their alternating counterparts (0/90° and 45°). All comparisons utilized tensile results at 25 °C as the reference point.

**Figure 9:**
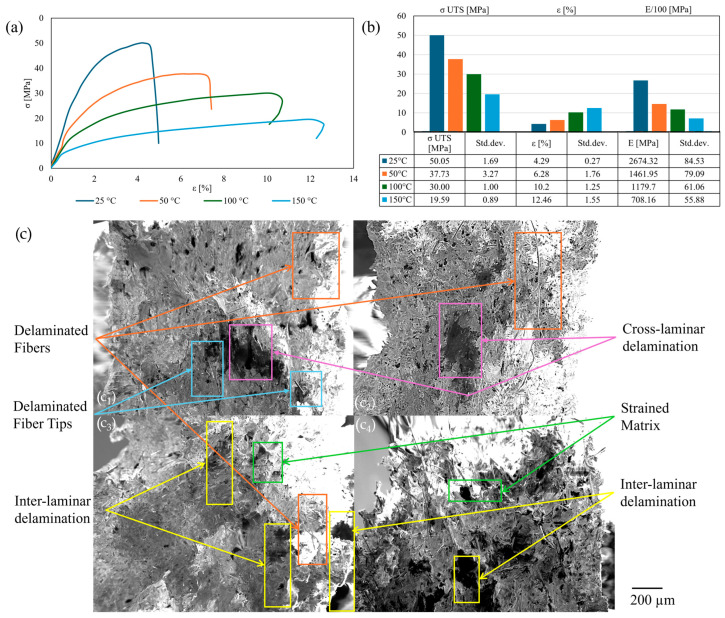
Thermal influence for 0° infill orientation: stress–strain curves (**a**); tensile results chart (**b**); cross-section SEM of the fracture area (**c**): 25 °C (**c_1_**); 50 °C (**c_2_**); 100 °C (**c_3_**); 150 °C (**c_4_**).

The initial bead configuration examined was 0° infill orientation. The stress–strain curves (Figure 9a) exhibited a brittle steep gradient profile at ambient temperature, which gradually transitioned into ductile-plastic curves above T_g_, as evidenced by a decrease in strength and an increase in plasticity (Figure 9b). Furthermore, the reduction in strain post-failure above T_g_ was attributed to the viscoelastic relaxation of the matrix under thermal influence. This was corroborated by a shift in the predominant fracture mode from cross-laminar with delaminated, deformed reinforcement to an inter-laminar failure mechanism, characterized by a strained matrix and delaminated reinforcement tips, as observed in the SEM images (Figure 9c). The tensile results indicated a peak σ _UTS_ of 50 MPa under ambient conditions, followed by a gradual decrease of 20% with each thermal increment. Regarding strain, T_g_ can be considered a pivotal point; values increased by 50% between the first and the last two increments, nearly doubling between 50 °C and 100 °C. The variation in Young’s modulus with temperature was reduced by half from the reference value upon reaching the first thermal increment, with a relatively minor further reduction of 10% over T_g_, followed by a subsequent 20% decrease at 150 °C.

**Figure 10:**
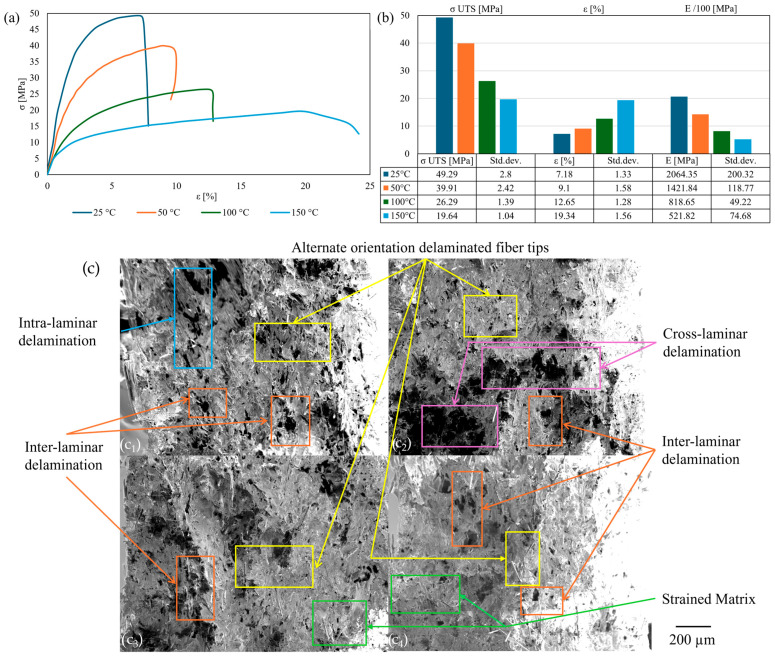
Thermal influence for ±45° infill orientation: stress–strain curves (**a**); tensile results chart (**b**); cross-section SEM of the fracture area (**c**): 25 °C (**c_1_**); 50 °C (**c_2_**); 100 °C (**c_3_**); 150 °C (**c_4_**).

The ±45° raster orientation presented similar results relative to the temperature stress–strain profiles (Figure 10a) as the 0° samples, confirmed by the tensile results (Figure 10b) and explained further by the SEM images of the fracture area (Figure 10c). It was observed that there was a gradual increase in ductility with each thermal increment, which was explained by the decreasing viscosity of the matrix. A post-failure strain reduction was observed at 50 °C, which can be attributed to the localized retraction of reinforcement, confirmed by the cross-laminar fracture. The increased ductile behavior from 100 to 150 °C is caused by the shift from cross-laminar/inter-laminar failure mode to the increased plasticity of the inter-laminar cross-links of the matrix over T_g_ [36]. In terms of tensile results variation with temperature, σ _UTS_ decreased by approximately 20% with the first thermal increment, losing half of its value above T_g_ (100 °C), and further by 20% by overcoming T_c_ (150 °C). The strain increased from 25% at the first thermal increment to 50% between 50 and 100 °C and almost doubled between the last two temperatures. Young’s modulus showed a steady decline of 30% with each thermal increment. The 45° specimens showed a similar tendency for thermal dispersion of the properties, with pronounced bead slipping resulting in a predominantly intra-laminar fracture mechanism.

**Figure 11:**
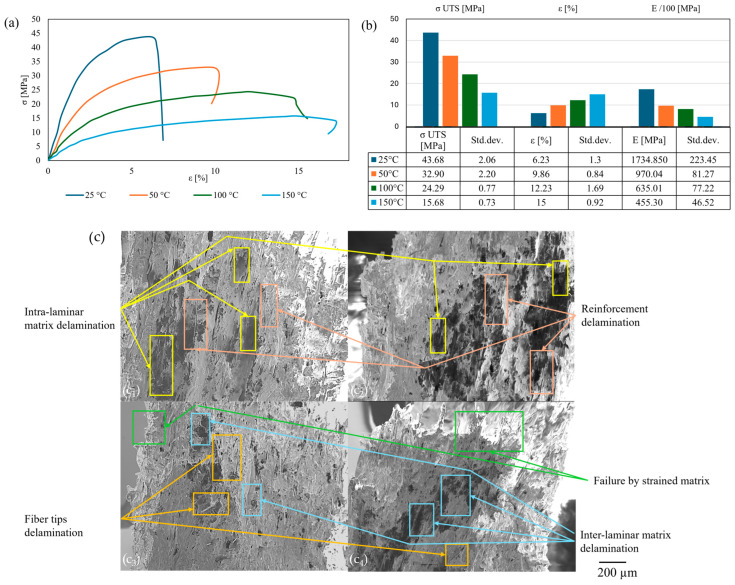
Thermal influence for 90° infill orientation: stress–strain curves (**a**); tensile results chart (**b**); cross-section SEM of the fracture area (**c**): 25 °C (**c_1_**); 50 °C (**c_2_**); 100 °C (**c_3_**); 150 °C (**c_4_**).

The tensile stress–strain curves (Figure 11a) for the least rigid infill orientation (90°) showed a gradual increase in ductility with temperature, confirmed by the tensile results (Figure 11b), with a similar behavior to the ±45° specimens at 50 °C, although a more pronounced strain post-failure was identified at 100 °C. This can be explained by the shift of the failure mechanism identified in Figure 11c from intra-laminar to a more plastic inter-laminar delamination, transitioning from delaminated reinforcement to a strained matrix with visibly delaminated fiber tips at 100 °C, and ultimately, fully embedded fibers in a strained matrix [34,35]. In terms of the variation in the results with temperature, σ _UTS_ and E decreased in a manner similar to that of the 0° specimens. The strain demonstrated a reduced variation compared with the other orientations, with a steady increase of 50% for each thermal increment. The 0/90° specimens exhibited similar behavior to their unidirectional components, showing a consistent inter-laminar principal failure mechanism.

To conclude, the ultimate tensile strength reduces by approximately 20–25% at 50 °C, whereas above T_g_, the degradation is roughly 40–45%, and at 150 °C, it decreases by about 60–66%. The strain increased by ≅25–60% at 50 °C, about 75–138% at 100 °C, and at 150 °C, ε varied between ≅140–170%. The Young’s modulus decreased by roughly 30–45% at 50 °C, about 50–60% at 100 °C, and ultimately ≅70–75% at 150 °C. Fracture mechanisms evolved from brittle intra-laminar or cross-laminar delamination to ductile inter-laminar strained matrix failure.

## 4. Conclusions

This study assessed the tensile behavior of 3D-printed thermoplastic composites over a broad thermal range (25–150 °C), emphasizing the optimal raster orientation. The 0° and ±45° configurations alternated as the strongest, whereas the 90° infill exhibited the lowest σ _UTS_. The influence of orientation on the ultimate tensile strength increased gradually, stabilizing above T_g_. The plasticity peaked for the 45° specimens, while the 0° orientation showed the lowest strain, with the effect of bead arrangement decreasing gradually above 50 °C. The maximum Young’s modulus was found for the 0° samples and the minimum for the 90° configuration. However, the variation across orientations remained relatively constant across all thermal conditions. Ambient values for the strongest specimens were compared to supplier data, showing 50% reductions in ε and σ _UTS_ and 30% in E. The failure mechanisms under T_g_ varied from brittle cross-laminar fracture (0°) to plastic intra-laminar (45°, 90°) or inter-laminar (±45°, 0/90°) delamination. Above T_g_, ductile matrix fracture was the principal breaking mechanism, accompanied by inter-laminar separation. The thermal evolution of strength showed a 20–25% strength degradation at 50 °C, maintaining approximately half its ambient value at 100 °C and one-third at 150 °C. The strain doubled above T_g_ and almost tripled at 150 °C. The Young’s modulus decreased by over one-third for the first thermal increment, stabilizing at a 20% decrease for each temperature increase. PAHT-CF demonstrated higher thermal stability than PLA [27] and is comparable to PEEK-CF [28], suggesting its potential for high-temperature cost-efficient applications such as tooling for conventional thermoplastic manufacturing. Further studies will focus on the effect of the supplier-recommended heat treatment on tensile performance using similar specimen configurations under the same thermal conditions.

## Figures and Tables

**Table 1:** PAHT-CF characteristics provided in the Technical Data Sheet. Adapted from [26].

No.	Property	Symbol	Value	Deviation	Unit
1.	Tensile Strength (planar)	σ_UTSXY_	92	±7	MPa
2.	Failure Strain (planar)	ε_xy_	8.4	±1.8	%
3.	Young’s Modulus (planar)	E_xy_	3860	±230	MPa
4.	Tensile Strength (*Z*-axis)	σ_UTSZ_	47	±5	MPa
5.	Failure Strain (*Z*-axis)	ε_z_	4.1	±1.2	%
6.	Young’s Modulus (*Z*-axis)	E_z_	2180	±130	MPa
7.	Glass Transition Temperature	T_g_	70	-	°C
8.	Crystallization Temperature	T_c_	140	-	°C

**Table 2:** Manufacturing parameters for composite tensile specimens. Adapted from [26].

No.	Parameter	Value	Unit
1.	Nozzle Diameter	0.4	mm
2.	Layer Thickness	0.2	mm
3.	Top/Bottom Layer Thickness	0.16	mm
4.	Relative Infill Rate	100	%
5.	Printing Speed	250	mm/s
6.	Infill Orientation	0°; 45°; 90°; 0/90°; ±45°	°
7.	Extrusion Temperature	290	°C
8.	Building Plate Temperature	100	°C
9.	Enclosed Printing Environment Temperature	45	°C

## Data Availability

The raw data supporting the conclusions of this article will be made available by the authors on request.
